# Confronting the challenge: a regional perspective by the Latin American pediatric infectious diseases society (SLIPE) expert group on respiratory syncytial virus—tackling the burden of disease and implementing preventive solutions

**DOI:** 10.3389/fped.2024.1386082

**Published:** 2024-07-31

**Authors:** Roberto Debbag, María L. Ávila-Agüero, José Brea, Helena Brenes-Chacon, Manuel Colomé, Rodrigo de Antonio, Alejandro Díaz-Díaz, Luiza Helena Falleiros-Arlant, Gerardo Fernández, Angela Gentile, Iván Felipe Gutiérrez, Daniel Jarovsky, María del Valle Juárez, Eduardo López-Medina, Abiel Mascareñas, Sebastián Ospina-Henao, Marco A. Safadi, Xavier Sáez-Llorens, Alejandra Soriano-Fallas, Juan P. Torres, Carlos N. Torres-Martínez, Claudia Beltrán-Arroyave

**Affiliations:** ^1^ Latin-American Vaccinology Society, Buenos Aires, Argentina; ^2^ Pediatric Infectious Diseases Division, Hospital Nacional de Roberto Niños “Dr. Carlos Sáenz Herrera”, Caja Costarricense de Seguro Social (CCSS), San José, Costa Rica; ^3^ Center for Infectious Disease Modeling and Analysis (CIDMA), Yale University, New Haven, CT, United States; ^4^ Facultad de Ciencias de La Salud del, Instituto Tecnológico de Santo Domingo, Santo Domingo, Dominican Republic; ^5^ Department of Epidemiology and Public Health, Hospital Pediátrico Dr. Hugo Mendoza, Santiago Domingo, Dominican Republic; ^6^ Executive and Scientific Director, Centro de Vacunación de Investigación (CEVAXIN), Panama City, Panama; ^7^ Pediatric Infectious Diseases Department, Hospital Pablo Tobón Uribe and Hospital General de Medellín, Medellín, Colombia; ^8^ Departamento de Salud del Niño de la Facultad de Medicina de la, Universidad Metropolitana de Santos, São Paulo, Brazil; ^9^ Department of Pediatrics and Infantil Surgery Oriente, Hospital Luis Calvo Mackenna and Faculty of Medicine, Universidad de Chile, Santiago, Chile; ^10^ Epidemiology Department, Hospital de Niños R. Gutiérrez, Universidad de Buenos Aires, Buenos Aires, Argentina; ^11^ Pediatric Infectious Diseases Department, Clinical Infantil Santa Maria del Lago, Bogota, Colombia; ^12^ Faculdade de Ciências Médicas da Santa Casa de São Paulo, Pediatric Society at São Paulo, São Paulo, Brazil; ^13^ Centro de Estudios en Infectología Pediátrica CEIP, Department of Pediatrics, Universidad del Valle, Clínica Imbanaco, and Grupo Quironsalud, Cali, Colombia; ^14^ Department of Pediatric Infectious Diseases, Hospital Universitario “José E. Gonzalez”, Universidad Autónoma De Nuevo León, Nuevo Leon, México; ^15^ Faculty of Medicine, Universidad de Ciencias Médicas, UCIMED, San José, Costa Rica; ^16^ Department of Pediatrics, Faculda de Ciências Médicas da Santa Casa de São Paulo, São Paulo, Brazil; ^17^ Clinical Research, Hospital del Niño Dr. José Renán Esquivel and Senacyt (SNI) y Cevaxin, Panama City, Panama; ^18^ Department of Pediatrics and Children Surgery, Universidad de Chile, Santiago, Chile; ^19^ Department of Pediatrics, Universidad El Bosque, Cafettor Médica SAS, Bogotá, Colombia; ^20^ Clínica El Rosario and Clínica del Prado, Faculty of Medicine, Universidad de Antioquia, Medellín, Colombia

**Keywords:** respiratory syncytial virus, long-acting monoclonal antibodies, nirsevimab, maternal vaccination, maternal RSV pre-F vaccination

## Abstract

Respiratory syncytial virus (RSV) is the leading cause of acute lower respiratory infections in children around the world. The post-pandemic era has resulted in a notable increase in reported cases of RSV infections, co-circulation of other respiratory viruses, shifts in epidemiology, altered respiratory season timing, and increased healthcare demand. Low- and middle-income countries are responsible for the highest burden of RSV disease, contributing significantly to health expenses during respiratory seasons and RSV-associated mortality in children. Until recently, supportive measures were the only intervention to treat or prevent RSV-infection, since preventive strategies like palivizumab are limited for high-risk populations. Advances in new available strategies, such as long-acting monoclonal antibodies during the neonatal period and vaccination of pregnant women, are now a reality. As the Regional Expert Group of the Latin American Pediatric Infectious Diseases Society (SLIPE), we sought to evaluate the burden of RSV infection in Latin America and the Caribbean (LAC) region, analyze current strategies to prevent RSV infection in children, and provide recommendations for implementing new strategies for preventing RSV infection in children in LAC region.

## 1 Introduction

Respiratory syncytial virus (RSV) is a leading cause of acute lower respiratory infections in children, representing a major public health concern globally. Country-specific RSV seasonality reports found that its epidemics usually start in countries from the Southern Hemisphere between March and June and in countries in the Northern Hemisphere between September and December (1–3). Post-pandemic, there has been a notable increase in reported cases of RSV infections and co-circulation of other respiratory viruses, accompanied by shifts in epidemiology, altered respiratory season timing, and increased healthcare demand (4, 5).

Latin America and the Caribbean (LAC) have encountered a parallel situation, intensifying the burden on an already vulnerable pediatric population struggling with socio-economic disparities, low education rates, limited access to medical care, lack of appropriate infra-structure for supportive management of severe cases, irregular migration status, and native communities limitations, among other challenges (6, 7).

Low- and middle-income countries, where most mortality from respiratory illnesses occurs, are responsible for the highest burden of RSV disease, contributing significantly to health expenses during respiratory seasons, both in outpatient and inpatient settings (8). The approach to treat RSV infection is primarily centered on supportive measures (9), and until recently, preventive interventions like palivizumab were limited for high-risk populations. However, despite the positive cost-effectiveness associated with preventive measures for premature newborns and infants with underlying conditions, no real global epidemiological impact to reduce burden of disease has been identified, mainly associated to difficulties up taking measurements like palivizumab due to high cost, difficulty of monthly administration, and financial discrepancies among countries in the LAC region (10).

Advances in new strategies for the prevention of infection with long-acting monoclonal antibodies during the neonatal period and vaccination of pregnant women are now a reality. In November 2022, the European Medicine Agency (EMA) approved nirsevimab, a long-acting, single-dose, monoclonal antibody for the prevention of RSV infection in newborns and infants, which was subsequently approved by the Food and Drug Administration (FDA) in August 2023. In the same year, the FDA also approved the use of an RSV preF vaccine for pregnant women, offering an alternative tool for the prevention of this global health concern (11). In LAC countries, these novel interventions have been approved in Brazil, Chile, and Argentina.

This review aims to evaluate the burden of RSV infection in LAC region and analyze possible strategies to prevent RSV infection in children, to accomplish a reduction of morbidity and mortality associated with respiratory infections and avoid the known deleterious effect of RSV in children's pulmonary health.

## 2 Methods

Given the global burden of RSV infection, various pharmacological preventive tools have been extensively researched. The regional expert group of the Latin American Pediatric Infectious Diseases Society (SLIPE), started in 2023 an analysis of the current published information regarding RSV preventive measures for infants, including long-lasting monoclonal antibody and vaccination for pregnant women. Literature including data from pre-clinical studies to phase III and IV clinical trials, to recommendations of international societies of the different measures where analyzed. After a period of individual review of available literature, the expert group conducted a series of meetings that lead to this document with an overview of the disease, highlights of different strategies, and a series of recommendations for implementing new strategies for preventing RSV infection in the LAC region.

## 3 Burden of RSV disease in Latin America and the Caribbean

Several indicators can measure the impact of RSV infection in children, including morbidity, hospitalization rates, direct and indirect costs, and other related variables in patients and families (1, 2). In 2019, the estimated global rate of LRTI caused by RSV was 48.8 cases per 1,000 children under 5 years of age, with approximately 33 million cases annually. Notably, 20% of these cases occur in infants under 6 months of age, particularly in low- and middle-income countries (LMICs) (6).

Despite higher incidence in the community, the RSV-associated LRTI hospital admission rate in LMICs was similar to that in high-income countries for children aged 0–60 months, and for children aged 0–6 months. Also, hospital admission rate in LMICs was consistently lower than that in high-income countries (6).

In 2019, more than 100,000 of the 52 million deaths due to all causes were attributable to RSV in children younger than 5 years of age, with higher proportion in those younger than 6 months of age (6). Mortality rate linked to RSV infection is directly correlated with economic income, especially affecting those under 1 year of age. Alarmingly, more than 97% of these deaths occur in low- and middle-income countries, with almost 80% occurring outside hospital settings (6).

There are widespread differences in the incidence of the disease, hospitalizations, and mortality can be attributed to various factors, including prevention policies, inadequate surveillance, underreported cases, difficulties in accessing medical attention, and resource availability in hospital settings.

The economic burden of RSV disease exhibits significant variations between countries. A systematic review by Zhang et al. (8) revealed that, for hospitalization in high-income countries, costs can range from 850 to 33,385 USD, with a mean length of hospitalization of 3 days (95% CI 3.03–3.15). In middle-income countries, these costs decrease to 87–634 USD per episode, with a length of stay of 6.4 days (95% CI 6.36–6.46). Costs for patients without hospitalization vary between 254 and 1,131 USD in high-income countries and 63 to 334 USD in middle-income countries. Interestingly, African, and LAC data were not included in this analysis (8). Another systematic review on the economic burden of RSV infection in middle- or high-income countries (including China, Malaysia, Mexico, and Colombia) during 2022, reported total costs ranging from 709 to 3,815 USD. Although the main causes of expenses lack consensus, it is established that treatment represents almost 30% of the total price of medical care (12).

In LAC, a systematic review conducted in 2022 identified high incidence rates of RSV infection in the region. Argentina reported a rate of 47.5 cases per 1,000 children per year, and Panama documented an incidence of 52.2 cases per 1,000 children (4). In Argentina, by 2019, it was observed that more than 60% of hospitalizations were in children younger than 12 months of age (35.3% in less than 6 months, 26.9% in 6–12 months, 19.5% in 12–24 months, and 18.1% older than 24 months), with similar findings in other LAC countries (Table 1) (6, 13, 14).

**Table 1:** Incidence rates of RSV-infection in Latin American countries.

Country	Incidence (per 1,000 children per year)	Number of episodes
Argentina	47.5 (35.1–64.4)	177,889 (131,258–241,085)
Costa Rica	47.6 (35.1–64.5)	16,705 (12,326–22,640)
Chile	47.7 (35.2–64.7)	56,448 (41,651–76,501)
Mexico	48.0 (35.4–65.1)	530,334 (391,316–718,739)
Bolivia	48.5 (35.8–65.7)	57,480 (42,412–77,900)
Colombia	48.7 (35.9–66.0)	181,578 (133,981–246,085)
Ecuador	48.9 (36.1–66.3)	81,335 (60,014–110,229)
Brazil	49.2 (36.3–66.7)	717,437 (529,373–972,311)
Panama	52.2 (38.5–70.7)	20,293 (14,974–27,503)

Adapted from: Li et al. (6).

Outcomes of disease exhibit variations based on age, revealing elevated admission rates to Pediatric Intensive Care Units (PICUs) in healthy children below 2 years old (14, 15). A Colombian trial reported a severity rate of 17.2%, identifying risk factors for ventilatory failure, including age younger than 6 months (OR: 4.58; 95% CI: 1.06–19.79; *p* = 0.041), low income (OR: 1.78; 95% CI: 1.06–2.99; *p* = 0.028), and malnutrition (OR: 3.99; 95% CI: 1.25–12.72; *p* = 0.019) (16), as observed in other LAC cohorts (14, 15). Mexico reported PICU admission rates ranging from 3.9% to 4.8% of all hospitalized cases (15, 17). In Argentina, it has been identified that 71.8% of all LRTI admissions to PICU were attributed to RSV (14).

The COVID-19 pandemic and the preventive measures (lockdowns, use of masks, and intensive hand hygiene) had a direct impact on respiratory virus circulation, including RSV (18, 19). In 2020–2021, a significant decrease in incidence, hospitalization, and mortality occurred in children under 5 years worldwide (20, 21). By 2021–2022, when most countries began withdrawing measures, a shift in seasonality was observed worldwide, with 2022–2023 cases being more comparable to previous seasons, yet respiratory infections season globally commenced earlier than anticipated (4, 22). Between 2019 and 22, the Public Health Institute in Chile documented RSV positivity rates of 25.2%, 0.2%, 10.3%, and 27.2% per year, respectively, aligning with global observations of viral circulation (23). This altered seasonality impacted the implementation of immune prevention strategies, resulting in higher hospitalization rates among high-risk patients (24). Also, an increase in age of affected children and the possibility of long-term sequelae associated has been documented, showing the need on availability of preventive measurements (25, 26).

## 4 Advances in preventive measures and perspectives for Latin America

Palivizumab (Synagis, Sobi, Inc, United States), a monoclonal antibody produced by recombinant DNA technology, was approved in 1998 for preventing severe LRTI due to RSV infection in high-risk pediatric populations, with proven safety and tolerability (27, 28). A multicenter observational study in LAC from 2011 to 2012 reported low RSV-related hospitalization rates in high-risk infants who received palivizumab according to routine clinical practice, endorsing the effectiveness and safety of palivizumab prophylaxis in the region (29). Nevertheless, this strategy has limitations, including high cost and limitations in real scenarios to complete all doses required of the monoclonal therapy to complete immunization. Switch to new preventive tools is recommended, but while countries acquire new measures, use of palivizumab is still recommended for high-risk infants.

### 4.1 Nirsevimab

Nirsevimab (Beyfortus, Sanofi y AstraZeneca) is a long-acting, single-dose, humanized monoclonal antibody class IgG1κ directed against the antigenic Ø site of the RSV F protein. It blocks virus entry, demonstrating higher affinity and neutralizing effect when compared with palivizumab. Due to Fc region modification, nirsevimab provides protection for at least 5 months, and use has been approved by entities around the world (30, 31).

Across all clinical trials the use of nirsevimab in infants was well tolerated in general, with a favorable safety profile. The overall rates of adverse events were comparable between nirsevimab and placebo and most adverse events were considered mild or moderate in severity. Cost of nirsevimab is lower than its predecessor, and based on early experiences, if coverage is high, the impact will probably be seen early in the intervention.

The implementation of an immunization strategy with nirsevimab, including all newborns and young infants, could provide substantial impact on the disease burden during their first RSV season. The extent of the reduction in disease severity relies on the duration of protection and the careful design of intervention campaigns specific to each country (32), and seasonality will also have an impact in nirsevimab use based on the protection of one dose. There is a growing need in genomic surveillance of RSV and the monitoring of emerging mutations that could impair the efficacy of these measures (33).

Use of nirsevimab is dictated by seasonality, presenting a challenge for global standardized recommendations, particularly in LAC with its varied latitudinal characteristics and seasonal patterns (34, 35).

Preliminary data of the introduction of nirsevimab in Spain documented that after a 3-week hospital-based immunization campaign, they reached a 97.5% uptake in high-risk groups, 81.4% in catch-up groups, and 92.6% in infants born during campaign, with a successful implementation strategy for the application of treatment (36, 37). A report from Luxemburg of the impact of nirsevimab prophylaxis during the initial 2023–2024 season when compared with 2022–2023, has documented an increase in age of hospitalized children due to RSV infection, a decrease in hospitalizations among those that received nirsevimab, with shorter lengths of hospitalization, and an apparent decrease in PICU admissions (38).

In LAC region, Chile is the first country to acquire nirsevimab for the use in all infants younger than 6 months of age, and implementation will begin in March 2024 (39). Immunization in Chile will begin in maternities around the country, and will continue with those younger than 6 months of age (40, 41).

The use of nirsevimab also rise challenges that need to be addressed by countries around the world. Cost/effectiveness studies and feasibility of immunization programs will be needed to guide recommendations from National Immunization Technical Advisory Groups (NITAGs) of the countries (32).

A theoretical possible change in the antigenic site where nirsevimab binds has been mentioned. A study analyzing genotypic changes in RSV after the administration of nirsevimab documented that no resistance-associated of clinical importance were identified, nevertheless, continued genotypic surveillance will be needed once nirsevimab is introduced in wider populations (42). A summary of the characteristics of the new long-acting monoclonal antibody as well as potential challenges for tits implementation in immunization programs is presented in Table 2.

**Table 2:** Characteristics of nirsevimab and maternal RSV vaccine and challenges of their introduction.

Characteristics	Challenges
1. Nirsevimab	
Easy administration with only one dose. Immediate protection, and protection for at least 150 days after administered. Expected good acceptance. Possibility of protection in a second respiratory season in children with underlying diseases. Flexible immunization schedule. Protection to premature infants. No known interaction with other vaccines. Lower cost than previous monoclonal antibody. Consistent neutralizing antibodies received after immunization. Prevention of severe RSV disease during early infancy, the period of highest risk of severe RSV disease and mortality Effective against both RSV A and B subtypes. Safety and reactogenicity comparable to WHO recommended vaccines given at the same age. A single dose can be given as a birth dose or at any healthcare visit during the first 6 months of life.	Cost Need for widely available genomic surveillance to identify potential antigenic or epitope change. Universal immunization schedules are not possible given the heterogeneity of respiratory seasons in LAC region. Limited documented experience with its use. Availability of prophylaxis may be limited for all countries around the world. Universal strategies are needed to avoid pragmatic application errors.
2. Maternal RSV vaccine	
Documented efficacy reducing infection in the first 180 days of life. Polyclonal response to maternal vaccination may reduce the possibility of resistance. Lower cost than monoclonal antibody.	Universal immunization schedules are not possible given the heterogeneity of respiratory seasons in LAC region. Logistic issues on acceptancy by the community and prescribing health providers. No flexible immunization schedule during pregnancy. Lower maternal vaccination coverage in several countries could impact the implementation of RSV vaccine. No protection for premature infants born before 34 weeks of gestation. Limited documented experience with its use. Neutralizing antibodies received by the newborn after maternal immunization may be unpredictable in some obstetric conditions.

### 4.2 Maternal RSV preF vaccination

RSV preF vaccine (Abrysvo, Pfizer Inc.) is a bivalent recombinant stabilized prefusion F protein subunit vaccine. Studies supporting its approval documented at 6 months after immunization, efficacy against medically attended RSV-associated LRTI and RSV-associated hospitalization: a 51.3% (29.4%–66.8%) efficacy against LRTI caused by RSV, and 56.8% (10.1%–80.7%) against hospitalization due to RSV-associated LRTI (34, 43). In the phase 3 randomized trial involving 7358 participants, vaccination during pregnancy has reduced the risk of severe RSV lower respiratory tract infection in infants by 82% within 90 days, and by 69% within 180 days (44). RSVpreF vaccine administered during pregnancy was effective against medically attended severe RSV-associated lower respiratory tract illness in infants, and no safety concerns were identified. Although not reaching statistical significance, a higher incidence of preterm births was noted among those who received the maternal RSV vaccine during weeks 24 through 36 of pregnancy compared to those given a placebo. Current available data do not provide adequate evidence to confirm or refute a direct link between the RSVpreF vaccine and preterm births. To minimize the risk of preterm births associated with the maternal RSV vaccine, the FDA has recommended its administration during the 32nd to 36th weeks of pregnancy. Nevertheless, EMA differs on recommendation for gestational age at which RSV vaccine can be administered. Also important, the study did not involve participants who were at an increased risk of preterm births, or high-risk pregnancies of any kind (45). Emerging data from high-income countries, as well as LMIC, are providing evidence that maternal vaccination or long-acting monoclonal antibodies are not only cost-effective, but potentially also cost-saving strategies (43, 46). High maternal vaccination rates are needed for immunization strategies to be successful, including RSV vaccine, so implementation of strategies in countries need to focus on this matter.

In LAC, Argentina has included maternal RSV vaccination in the national vaccination schedule to be mandatory and free since 2024. In accordance to current 2024 PAHO recommendation (45), previously in 2023, Argentina recommended the use of maternal vaccination between weeks 32 and 36 of pregnancy during the RSV season. Long-acting monoclonal antibodies would be available for premature infants younger than 32 weeks' gestation, those born from mother that did not received vaccination, and infants with congenital heart and lung disease using current palivizumab recommendation (47).

As shown in Table 2, maternal vaccination has several characteristics that anticipate its benefits, specially related to cost, efficacy, and response to vaccine application.

Nevertheless, several challenges need to be explored before introduction of vaccination. Most recent US estimates on maternal vaccination rates, showed that in 2019–2020, only 61% of pregnant women received influenza vaccine, 57% received Tdap, and 40% both (48). By 2018 of the 49 countries from LAC territories, only 32 offered vaccination against influenza to pregnant women and 29 provided tetanus-containing vaccine (49). While countries such as Argentina have been able to accomplish adequate vaccination coverage during pregnancy (50), other countries like Colombia have struggled with vaccination coverage, especially after the COVID-19 pandemic (51). Differences in vaccine coverage in the region will impact the introduction of maternal RSV vaccination.

Until more information is available, concerns related to RSV maternal vaccination and prematurity may impact acceptance and recommendation of this strategy among pregnant women and health providers. Also, RSV vaccination may reduce immunogenicity of Tdap when administered simultaneously, creating a challenge for the introduction of RSV maternal vaccine in national immunization schedules.

## 5 Expert regional recommendations

RSV infection poses significant morbidity and mortality in children, especially among vulnerable groups, and it is associated with bacterial invasive infections, excessive antibiotic use, increased prescription of bronchodilators and steroids.

Based on the detrimental impact of RSV infection on medium- and long-term pulmonary health, the Regional Expert Group of the Latin American Pediatric Infectious Diseases Society (SLIPE) provides expert-based recommendations for implementing new strategies for preventing RSV infection in children in LAC region.

1. Health authorities and decision makers in LAC region are strongly encouraged to adopt preventive strategies against RSV infection. These involve the implementation of vaccination against RSV during pregnancy, use of long-acting monoclonal antibodies, or a combined strategy (vaccination during pregnancy and availability of monoclonal antibodies for those born from non-vaccinated women as well as premature infants)
2. Although better information is still needed from determined countries, the current available evidence in the LAC region of the burden of RSV disease in children is sufficient to support recommendations of implementing new preventive strategies.
3. It is advisable to implement or reinforce epidemiological surveillance systems, encompassing the study of genomic characteristics of the virus, and assess the impact of these strategies once implemented. Despite potential challenges in local data availability, these interventions should be swiftly adopted, emphasizing the urgency of their implementation, and follow-up of generated results.
4. Encouraging communication between the scientific community and the general public is essential, with particular attention to the behavior of pregnant women, adolescent mothers, but also families in general. This communication is crucial for fostering understanding and adherence to preventive measures.
5. Evidence generated regarding the impact, effectiveness, and safety of these preventive measures, with a uniform methodological analysis, should be shared across the region. Following the implementation of preventive strategies, ongoing surveillance of safety and post-introduction impact, along with identifying limitations, will assist countries in dynamically improving their chosen measures.
6. For LAC region, nirsevimab is recommended for all infants younger than 6 months, whose mothers did not receive RSV vaccine during pregnancy, and who are born during or entering their first RSV season, and for infants and children aged 8–19 months who are at increased risk for severe RSV disease and are entering their second RSV season.
7. Maternal vaccination is recommended in all pregnant women between 32 and 36 weeks of gestation. Preterm infants born before 32 weeks, or those born in the first two weeks after maternal vaccination, should receive nirsevimab as recommended.

## 6 Conclusions

RSV infection stands as the leading cause of LRTI in children. It is important to emphasize that, despite the presence of well-known risk factors associated with more severe RSV infection, 70%–80% of hospitalizations and deaths associated with RSV occur in previously healthy, full-term born children. Recent approval of new preventive measures, such as a single-dose long-acting monoclonal antibody and maternal RSV preF vaccination, represents a unique opportunity to at last effectively prevent severe RSV-LRTI in infants, reducing the dramatic burden associated with this infection in infants. Given the worldwide prevalence of RSV infection, implementation of these strategies must be *a priori*ty in all countries, particularly in LMIC from LAC, where RSV represents a major public health concern. Equitable access to these new strategies for the prevention of severe lower respiratory tract infection caused by respiratory syncytial virus in infants is a clear demand. In this context, improving RSV surveillance in LAC countries, with quality information, will be of paramount importance to inform future implementation of immunization programs in the region.

## Data Availability

The original contributions presented in the study are included in the article, further inquiries can be directed to the corresponding author.
